# Effect of Coffee and Polishing Systems on the Color Change of a Conventional Resin Composite Repaired by Universal Resin Composites: An In Vitro Study

**DOI:** 10.3390/ma16176066

**Published:** 2023-09-04

**Authors:** Gözde Aksoy Vaizoğlu, Nuran Ulusoy, Laden Güleç Alagöz

**Affiliations:** 1 Department of Restorative Dentistry, Faculty of Dentistry, Near East University, Mersin 10, Nicosia 99138, Turkey; nuran.ulusoy@neu.edu.tr; 2 Department of Restorative Dentistry, Faculty of Dentistry, International Final University, Mersin 10, Girne 99320, Turkey; laden.alagoz@final.edu.tr

**Keywords:** universal composite resins, polishing systems, color measurement

## Abstract

The purpose of this study was to evaluate the color stability of repaired aesthetic restorative resin matrix materials after immersion in coffee and the effect of polishing systems after staining. One hundred and eighty cylindrical discs (8 mm × 2 mm) were prepared using a conventional nano-fill resin composite (Clearfil Majesty Esthetic A2 shade) with round cavities (3 × 1 mm). Cavities were repaired by three resin composite materials: Clearfil Majesty Esthetic A2 shade, one-shaded nano-fill resin composite (Omnichroma) and group-shaded nano-hybrid resin composite (Optishade, medium shade). Each group was polished with three polishing systems (n = 20); aluminum oxide (Soflex Spiral Wheels, 3M ESPE), silicon carbide (Occlubrush, Kerr, CA, USA) and diamond particulate (Twist Dia Spiral Wheels, Kuraray, Okayama, Japan). Color change (ΔE_00_) measurements were performed with a spectrophotometer at the baseline. Half of the polished samples were either kept in distilled water or immersed in coffee for 15 days, and color measurements were repeated before and after polishing. Statistical analysis was performed using the Kruskal–Wallis test. Repaired samples showed different color correspondence values in all groups. All three restorative materials showed significant color changes (ΔE_00_) after immersion in coffee (*p* ≤ 0.05). Repolishing of stained samples showed color improvement values in all groups. The content of the polishing system played an important role in removing the stains.

## 1. Introduction

Over the past 10 years, improvements have been made in the esthetic properties of resin composite materials in response to the increasing esthetic expectations of patients and the demands of clinicians for materials with optical properties similar to those of natural teeth [1,2]. Recent resin composites are available in a wide range of colors and are able to reflect the colors of the surrounding dental tissue [3]. 

It is generally known that the filler (type), size range, and volume content have a major impact on the mechanical properties of resin composites [4]. The small size of the filler particles enhances the optical characteristics of resin composites. Nowadays, nanocomposites and supra-nanocomposites are used for both anterior and posterior restorations because of their superior clinical performance in terms of both functionality and esthetics [5]. Given the countless options for colors and opacities that can be employed to simulate the optical characteristics of dental structures, the initial result of a direct restoration may be outstanding [6].

Clinical indications such as secondary caries, marginal defects, marginal staining, superficial color correction, wear resistance of the resin composite, bulk fracture, or fracture of an adjacent tooth may lead to resin composite repair [7]. In accordance with the philosophy of “minimal intervention” dentistry, in dental practice, restoration repair is preferred to increase the survival rate of the restoration instead of removing it completely [8]. Along with the economic reasons, the aim of preserving as much healthy dental tissue as possible and/or preventing pulpal symptoms may be the reason for preferring the repair procedure [2]. However, we may not always know the brand and type of resin composite used in preexisting restorations; thus, in this case, the properties of the material to be selected for the repair are important for increasing the quality of the procedure [9].

Today, universal resin composites include supra-nanoparticles and are produced using the sol–gel method, which controls the refractive index of fillers. Manufacturers provide data showing that spherical fillers maintain high gloss retention and provide opalescence by reflecting light (chameleon effect). Spherical fillers are mathematically lined up from bottom to top homogeneously. This particular line model provides an easier repair process and naturally good-looking restorations [10].

The tendency to simplify shade selection has resulted in the development of universal composites, since color selection can be difficult and dependent on environmental and operator-dependent variables. Recently, a composite material with a single hue that purportedly matches all Vita Classical shades, from A1 to D4, was created. The chameleon effect of resin composites reduced the number of shades. In recent years, supra-nano spherical filler composites called ‘Omnichroma’ have been developed. These materials produce more color stability in a single shade [11]. In addition, ‘Optishade’ resin composite, which is a universal nanocomposite shade, is available on the dental market. A literature study that compared the color adjustment potential of a universal resin composite, a group-shade resin composite and a multi-shade resin composite found that, universal resin composite had a significant best result [12]. Developers advise the use of these materials in a single-shade increment to possibly match varied tooth colors because they have a universal opacity and a limited number of Vita shades [3]. Environmental light illuminance on a color has the most important effect on measurements. The light must be standardized and given a relative visual performance. A literature study that compared the CIE standard 6 light sources found that the D65 light source is the best choice. Accordingly, color parameters were measured using a spectrophotometer under ISO light D65 as a standard [13].

Finishing and polishing procedures are crucial steps that improve the esthetics and durability of repaired restorations by preventing plaque retention and staining, which will ultimately affect the quality, surface integrity, and surface roughness [14]. Various methods are available to finish and polish resin composite restorations. The majority of the literature indicated that polishing systems with aluminum oxide were the gold standard on micro-filled resin composites. However, diamond particulate polishing systems had a significant effect on packable and hybrid resin composites [15]. Nowadays, most clinicians currently use polishing systems with silicon carbide and diamond particles. These systems require one or more processes and vary substantially in terms of the type of composition and hardness of the abrasive particles they include [16]. According to developments, one-step finishing and polishing wheels with pure diamond particles have been produced [17]. A review on the literature on polishing systems reported that micro-filled composites showed high-gloss surfaces with aluminum oxide polishing systems such as Soflex Disc or Astropol, but nano-filled and supra-nano-filled resin composites tended to show high-gloss surfaces with diamond polishing systems such as Soflex Spiral Wheels or Twist Dia [18].

In addition to the developments in polishing systems, improvements in color stability have been achieved by differentiating the content of resin composites. Color stability, which is one of the most important disadvantages of resin composites, has been brought under control by changing the composite resin content. With the improvement of nanofilament resin composites, the disadvantages that may occur on color stability have been minimized. In addition to these resin composites, there are resin composites produced by adding particles such as e-glass fibers, barium glass, and silicon dioxide. There are studies that support this issue in the literature. A study that evaluated the fiber-resin-reinforced composite materials according to cavo-surface marginal discoloration found that there was no significant discoloration between first measurements and either 6-month or 1-year measurements. The study reported that when compared conventional nano-hybrid resin composites regarding discoloration and noted gradual discoloration over time [19]. 

Despite appropriate color selection, external factors, repairs, and polishing can affect restoration aging. Nowadays, coffee consumption is comparatively higher than in previous times. The study aims to evaluate the color stability of a conventional resin composite repaired by the same conventional resin composite and two different resin composites along with the effectiveness of three different polishing systems in removing the discoloration after immersion in coffee. 

Moreover, to the authors’ knowledge, there are no studies evaluating the effectiveness of novel finishing and polishing wheels on conventional resin-based restorations repaired with one-shade and group-shade resin composites before and after staining with coffee. 

The research hypotheses tested were as follows:

I. After the repair procedure, the polishing systems with different abrasives would cause different results on resin composites;
II. Coffee would affect the color change of repaired restorations;
III. After the staining process with coffee, novel polishing systems would have an impact on the color change of universal resin composites.

## 2. Materials and Methods

### 2.1. Preparation of Specimens

This study investigated three different resin composites (conventional multi-shade (Clearfil Majesty Esthetic, Kuraray, Okayama, Japan ) group-shade universal resin composite (Optishade, Tokuyama Dental, Encinitas, CA, USA), one-shade universal resin composite (Omnichroma, Tokuyama Dental)) and three different polishing systems: aluminum oxide particulate (Soflex Spiral Wheels, 3M ESPE, St. Paul, MN, USA), silicon carbide particulate (Occlubrush, Kerr, CA, USA), and diamond particulate (Twist Dia Spiral Wheels, Kuraray, Okayama, Japan). A seventh-generation single-component self-etching adhesive and coffee as immersion material were used (Table 1). 

A total of 180 cylindrical discs (8 mm diameter and 2 mm depth) were prepared as base material with an A2-shaded conventional resin composite (Clearfil Majesty Esthetic, Kuraray, Okayama, Japan) using a metal mold. The mold was placed on a piece of glass with a transparent mylar strip on it, filled with uncured resin and then covered with another transparent mylar strip and glass. Each specimen was light-cured for 20 s using a LED Light curing unit (LCU) (Woodpecker, Led B, Curing Light, Shenzhen, China) with a light intensity of 1000 mW/cm^2^. The cylindrical discs were immersed in distilled water for 24 h after polymerization. 

Round-shaped cavities opened with a diamond burr under water conditions with a high-speed instrument 3 mm in diameter and 1 mm in depth were prepared on the cylindrical discs, and 3 × 1 mm metal molds were used to mark the discs for standardization. The specimens were divided into three groups (n = 60). 

### 2.2. Specimen Repair

**Figure 1:**
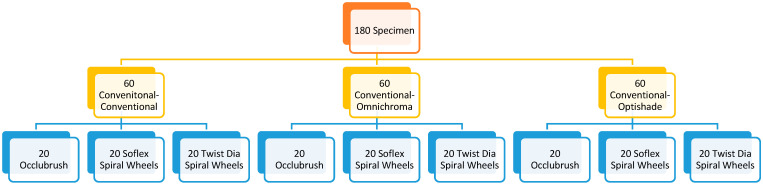
Study Groups.

The prepared specimens were either repaired with conventional resin composite, one-shade resin composite or group-shade resin composite. Based on the resin composites, the specimens were divided into three groups as shown in Figure 1.

Group 1. Clearfil Majesty Esthetic A2 Shade (C): 60 specimens were repaired by the same conventional resin composite. 

Group 2. Omnichroma (OMNI): 60 specimens were repaired with one-shade resin composite. 

Group 3. Optishade (OPTI): 60 specimens were repaired with group-shade resin composite of medium-shade.

Resin-composite materials were dispensed in a dark room and applied with a plastic instrument and condenser. The specimens were standardized, as the cavity dimensions were similar and the material placement was the same for all specimens. After packing, a mylar strip was applied and the resin composite was cured for 20 s with a LED light-curing unit (Woodpecker, Led B Curing Light China). After the curing process, the specimens were kept in distilled water for 24 h at 37 °C, until polymerization of the resin composite was complete. All restorations were performed by the same operator. 

### 2.3. Specimen Polishing

**Figure 2:**
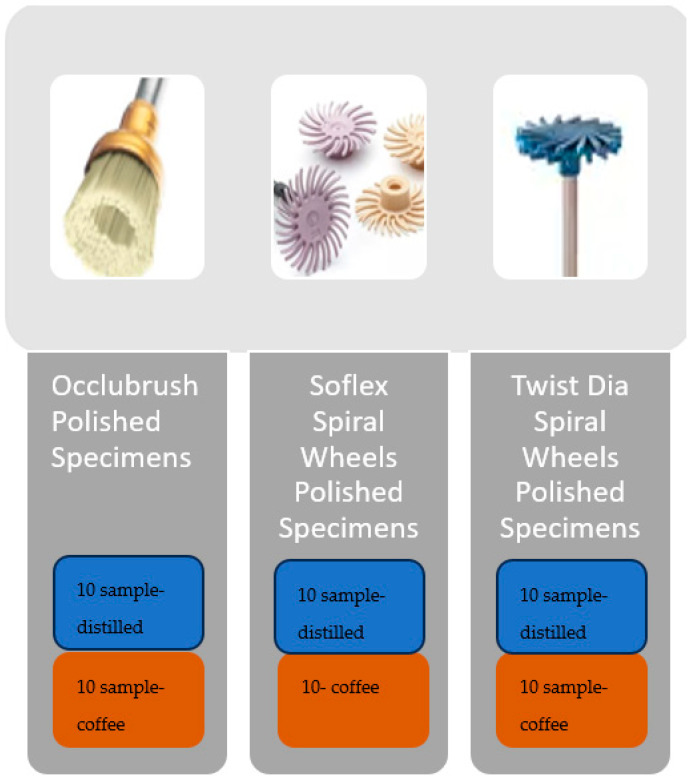
Staining procedure for each group.

Each restored group was divided into three subgroups (n = 20) and polished with Occlubrush (Kerr, CA, USA), Sof-Lex Spiral Wheels (3M Espe, USA) or Twist Dia Spiral Wheels (Kuraray, Okayama, Japan). Polishing was applied before and after the staining procedure to the specimens (Figure 2). Before the first measurement, polishing was applied on specimens as a control group. After immersion, polishing was applied as a repolishing procedure on specimens. 

Each sample was polished for 10 s under water cooling. The Occlubrush and Twist- Dia Spiral Wheels were changed in every three samples, whereas the Soflex Spiral Wheels were changed in every sample. All polishing procedures were carried out by the same operator. 

### 2.4. Staining Procedure

Staining procedures for each group are shown in Figure 2. Every polished group was divided into two groups (n = 10) and immersed in two different types of liquids, namely Nescafe (1 teaspoon of Nescafe Gold, USA, in 200 mL boiled water) and distilled water for 15 days under 37 °C. It has been reported that an average coffee drinker consumes 3.2 cups per day and spends 15 min drinking a single cup of coffee. Therefore, 24 h of coffee immersion simulates 1 month. Thus, storing a polished group in coffee for 15 days simulates approximately 1.3 years of coffee consumption according to the literature [14]. After 15 days, for standardization of the samples, the samples were removed from the cups, rinsed for 10 s (simulated saliva) to rinse away the excess coffee, and air-dried.

### 2.5. Color Assesment

The color or shade of the samples were measured respectively after being polished in the repairment procedure as the baseline (T0), after the staining process (T1) and after repolishing was applied to the stained specimens (both distilled water and coffee-stained specimens (T2)). All measurements were made in a viewing booth under D65 illumination using a spectrofotometer (Vita Easyshade Compact, VITA Zahnfabrik, Bad Säckingen, Germany). Prior to each measurement, the clinical spectrofotometer was calibrated according to the guidelines prescribed by the manufacturer, following which the probe was placed in the center of the sample surface. Measurements for each sample were repeated three times, and mean values were calculated for each specimen by the same operator. The background (5 cm × 2 cm) was created with A2-shaded conventional resin composite itself to simulate the color of the dark underlying dental structure [20]. The photometer was calibrated according to the National Institute of Standards and Technology (NIST) rules. The setting at which the tests were run included a 10 nm wavelength interval, 360 to 750 nm spectral range, and 45◦ reflectance angle. The color values of all samples were recorded according to the L*a*b* system created by the CIE Commission, where L*, a* and b* values represent lightness, the red-green-axis and the yellow-blue axis, respectively. The color change (ΔE_00_) of each sample was calculated using the CIEDE2000 formula [21].

$${\Delta E}_{00}=\sqrt{{\left(\frac{\Delta L^{\prime }}{k_{L}S_{H}}\right)}^{2}+{\left(\frac{\Delta C^{\prime }}{k_{C}S_{C}}\right)}^{2}+{\left(\frac{\Delta H^{\prime }}{k_{H}S_{H}}\right)}^{2}+RT\frac{\Delta C^{\prime }}{k_{C}S_{C}}\frac{\Delta H^{\prime }}{k_{H}S_{H}}}$$

The CIEDE2000 color difference formula in Microsoft Excel was used for this analysis, as previously proposed. Parametric factors were set to K_L_ = 2, K_C_ = 1, and K_H_ = 1. The parameters described in the literature were used to determine the threshold of perceptibility (PT) and the threshold of acceptability (AT). The CIEDE2000 (ΔE_00_) values in dentistry were 0.8 for 50:50% PT and 1.8 for 50:50% AT. Thus, the color change was considered undetectable when ΔE_00_ ≤ 0.8 and considered clinically unacceptable when ΔE_00_ ≥ 1.8 [22].

### 2.6. Statistical Analysis

Statistical analysis was carried out using the package for the social sciences (SPSS) version 20 (IBM, Armonk, NY, USA) as statistical software, and the significance level was set at *p* ≤ 0.05, since parametric assumptions were not provided and more than two group comparisons were made. The Kruskal–Wallis Test was used as a non-parametric statistical method in this study. The variables obtained by measurements are presented as mean ± standard deviations in tables in Section 3. 

## 3. Results

### 3.1. Color Stability

The present study evaluated the color stability ability of a single-shade (Omnichroma) and a group-shade resin composite (Optishade Medium Color) against a conventional restorative resin material (Clearfil Majesty Esthetic A2 Shade) after coffee immersion. The Kruskal–Wallis Tests showed significant differences among the study groups. The ΔE (color difference) values for the groups were compared one by one, as shown in Table 2, Table 3 and Table 4. As a result, coffee immersion affected the color of resin composite samples. A comparison of universal resin composites against conventional resin composites showed that one-shade resin composite was the material most affected by staining.

The calculated delta values between polishing types and different immersion solutions were analyzed, as shown in Table 2. While the average value of the measurements of specimens kept in distilled water was 1.80 ± 0.35, the measurements of specimens kept in coffee was higher, with an average of 9.82 ± 1.16. A statistically significant difference was found between the first measurement times (T0–T1) of polishing by Occlubrush in the two immersion solutions. The T0–T1 and T0–T2 measurements of polishing with Twist type in different immersion solutions showed a similar distribution between the two immersions. In the measurements of specimens kept in distilled water, only the T1 and T2 measurements were found to be different from each other. For specimens kept in coffee, differences were found for all three measurement times.

The calculated delta values between polishing types and different immersion solutions were analyzed in Table 3. A statistically significant difference was found between the first measurement times (T0–T1) of the Occlubrush polishing type in different immersion solutions. While the average value of the measurements of specimens kept in distilled water was 2.70 ± 2.01, the measurement of specimens kept in coffee was higher, with an average of 15.9 ± 1.98. The T0–T2 measurements of the Twist polishing type and the T0–T2 measurements of the Soflex polishing type in different immersions showed similar distributions between the two media. They were not found to be statistically different. At the same time, the difference between polishing types was also examined. There was no difference between the measurements of specimens kept in distilled water and the T0–T2 measurements. For the measurements of specimens kept in coffee, Occlubrush polishings were found to be different from the other groups in the measurements T1–T2. For the T1–T2 measurements, the measurements of Twist polishing were found to be different from the other groups.

As shown in Table 4, the calculated delta values between polishing types and different immersion solutions were analyzed. A statistically significant difference was found between the first measurement times (T0–T1) of the Occlubrush polishing type in different immersion solutions. While the average value of the measurements of specimens kept in distilled water was 2.01 ± 1.10, the measurements of specimens kept in coffee was higher, with an average of 13.3 ± 2.83. The T0–T2 measurements of the Twist polishing type and the T0–T2 measurements of the Soflex polishing type in different immersion showed similar distributions between the two immersion solutions. They were not found to be statistically different. At the same time, the difference between polishing types was also examined. For the measurements of specimens kept in distilled water, a statistically significant difference was found between the polishes for all measurement times. For the measurements of specimens kept in coffee, a statistically significant difference was found between the polishes for all measurement times.

### 3.2. The Effect of Repolishing

The present study evaluated the effect of the repolishing ability of standard aluminum oxide polishing system Occlubrush against the new-generation silicon carbide polishing or diamond polishing after immersion. The Kruskal–Wallis Tests showed significant differences among the study groups. The ΔE (color difference) values for the groups were compared one by one, as shown in Table 5, Table 6 and Table 7. As a result of the repolishing procedure, the diamond particulate polishing system showed significantly better results than the others. 

As shown in Table 5, the delta values between the values obtained from different polishing types were calculated. Occlubrush–Twist differences in different immersion solutions showed similar distributions; while the average value of the measurements of specimens kept in distilled water was 1.75 ± 0.45, the measurements of specimens kept in coffee was found to be lower, with an average of 1.25 ± 0.28. At the same time, the difference between the measurement times was also analyzed. In the measurements in distilled water, the T2 measurement value was found to be different from the other groups in Occlubrush–Soflex values. The polished measurements were also found to be different for Soflex–Twist compared to the other groups.

As shown in Table 6, the delta values between the values obtained from different polishing types were calculated. A statistically significant difference was found between the Occlubrush–Soflex T2 measurements. Occlubrush–Twist differences in different immersion solutions showed similar distributions; while the average value of the measurements of specimens kept in distilled water was 4.02 ± 0.96, the measurement of specimens kept in coffee was higher, with an average of 10.9 ± 1.93. At the same time, the difference between the measurement times was also analyzed. In distilled water measurements, Occlubrush–Soflex and Occlubrush–Twist values showed similar distributions for all measurement times. Soflex–Twist values were lower in the T2 measurement against the other groups.

As shown in Table 7, the delta values between the values obtained from different polishing types were calculated. In the measurements in distilled water, Occlubrush–Twist values showed a similar distribution for all measurement times. T0 of Occlubrush–Twist and Soflex–Twist differences in different immersion solutions showed similar distributions. A statistically significant difference was found between the Occlubrush–Soflex T2 measurements. At the same time, the difference between the measurement times was also analyzed. Soflex–Twist values were found to be lower in the T1 measurement compared to the other groups.

### 3.3. Comparison of Color Correspondence among Resin Composite Groups

The present study evaluated the comparison of color matching among resin composite groups with respect to the effect of immersion in liquids and repolishing (Table 8). A comparison of one-shade and group-shade universal resin composite against conventional resin composite showed that one-shade resin composite was the material most affected by staining. Conventional–conventional repaired resin composite samples showed the best color correspondence in baseline measurements (T0) after polishing was applied with Twist Dia spiral wheels with diamond particles. 

As shown in Table 8. the calculated ΔE_00_ (color difference) values between first measurements before immersion and after immersion were compared according to the polishing type.

Group 1. The conventional resin composite-repaired specimens exhibited significant differences according to their measurement times, whereas the Occlubrush polishing system showed no significant differences in the mean values between the T1 and T2 measurement times after 15 days. In the Soflex polishing system, a difference was observed between T1–T2 values. The Twist polishing system showed a very significant mean value between T1 and T2 measurement times, and it also showed no significant difference between the T0 and T2 measurements.

Group 2. In the Omnichroma-repaired resin composite specimens, Occlubrush showed a significant mean value between the T0 and T2 measurements. Soflex and Twist did not show significant mean values between the T0 and T2 measurements.

Group 3. In the Optishade-repaired resin composite specimens, Occlubrush, Soflex, and Twist showed significant mean values between the T0 and T2 measurements.

As a summary, color adaptation after the repairment procedure with T0 measurement time and the other T1–T2 measurement times was compared. In Group 1, of conventional resin composite repaired by conventional composite resin, there was no polishing-type effect on color correspondence after first polishing (T0). However, after immersion, there was a significant effect on color correspondence through the Twist polishing system against the others. In Group 2, conventional resin composite repaired by Omnichroma resin composite, the Occlubrush polishing system-polished specimens showed the best color correspondence in the first time measurements (T0). However, after immersion (T1, T2), the Twist-polished specimens showed the best color correspondence. In Group 3, conventional resin composite repaired by Optishade resin composite, Twist-polished samples showed the best color correspondence in all measurement times (T0, T1, T2). 

The L, C, H values of measured samples are shown in Table 9. The C and H values are important for understanding the visual color against the ΔE values. ΔE values show irrelevant results, but when ΔH stands, yet ΔC changes, the direction of values will be changed and the observer can see it as a visual. But in this study, ΔE values are supported by ΔC and ΔH values.

According to these results, distilled water T0 values were control group measurements. In all groups, ΔC values showed a significant increase between T0–T1 measurement times. After repolishing of stained samples (T2), a decrease in values was observed. ΔH values of all samples decreased according to T0–T1 values but when the repolishing procedure was carried out (T2), little increase was observed in most of the groups. In the Occlubrush-polished group 1, ΔH values showed nearly the same results between T1–T2 measurement times.

## 4. Discussion

As problems occurred with visual methods, instrumental methods were developed to reduce these disadvantages. Spectrophotometry, Spectroradiometry and digital imaging are the most popular methods. For color measurements, we used CIE systems based on L, a*, b* parameters. Spectrophotometers based on L, a*, b* parameters were used to measure the color. In our study, we used the methods most used in the literature. While they have advantages, the edge-loss phenomenon is the disadvantages of this method. To counteract this disadvantage, we measured all specimens three times at the same time and averaged these data to obtain a reliable result [23,24,25,26,27].

The L*a*b* color space, created by the CIE in 1978, is used to record color parameters. This system is related to human color perception in all three directions or regions of the color space. A* and b* are the chromaticity coordinates, whereas L* is a lightness variable whose value is proportional to Munsell’s value [28]. A more complex formula, CIEDE2000, was created by the CIE in 2001 in an effort to improve the CIELAB formula which measures the difference between two colors. The literature that compares CIE Lab and CIE2000 formula suggests that the CIE2000 formula is more reliable than CIE Lab. According to this literature, observers give nearly the same results with CIEDE2000 formula, which means that the CIEDE2000 formula reflects the color differences nearly the same as the human eye [29,30].

The other most important factor is the base on which we can place the sample and measure the color. As a result of a study that compared four backgrounds (black, white, resin composite and porcelain), the black background was found to be more useful than other backgrounds in Class III-IV cavities, but for other cavities that also have backgrounds such as enamel or dentin, the resin composite background was found to be more useful [21]. In our study, the resin composite background was used to evaluate the color.

Shade-matching composites of smart chromatic technology recently gained popularity as smart monochromatic resin composites. The size of their filler particles determines how well they can capture the structural color from the surrounding teeth. They do not contain any additional dyes or pigments, whereas the fillers naturally provide a red-to-yellow structural hue that blends in with the surrounding teeth [31]. In addition, universal resin composites with increased color-adaptive potential make the repair process easier and less time-consuming. Consequently, in our research, cavities were opened on a conventional resin composite and two universal resin composites were used for repair to evaluate their effect on the color match.

There are studies in the literature which compare color matching between conventional and universal resin composites. A study examined the color matching of different shades (B1-B2-A3-C3) on lower molar acrylic teeth with 4 mm diameter and 2 mm depth cavities which were restored with Omnichroma and conventional resin composites. B1, B2, and A3 conventional resin composites showed better color matching than Omnichroma. However, Omnichroma showed the best color matching compared to the conventional resin composites with the C3 shade [32]. Another study that compared the color and translucency potential of three different universal resin composites with one conventional resin composite showed that Omnichroma has the best color matching potential [33]. In our study, conventional resin composite samples were repaired with either conventional resin composite or two universal resin composites and then polished with three polishing systems. According to the first measurement time, the conventional resin composite had the best color matching potential when repaired with itself. However, when repaired with Omnichroma, the lowest color matching values were obtained. These conclusions directly tell us that the best repairment material is the same material which was used first.

The effectiveness of the finishing and polishing methods has a significant impact on the durability and aesthetic attractiveness of tooth-colored dental restorative materials [34]. Improper polishing of the restoration may cause the biofilm to attach to the tooth surface. The null hypothesis ‘I’ was accepted, as the three different polishing systems showed different values. Three polishing systems with different abrasives showed different delta values in all measurement times. The aluminum oxide particulate polishing system showed the lowest delta values against the others, and the diamond particulate polishing system showed the best delta values after repolishing the specimens after immersion.

Several variables, including filler particle size, filler loading and type, resin amount and particle shape, affect the surface quality of the resin composite. When smaller particles were added to the resin composite materials, the polishing success was significantly improved [18]. Due to their small particles and high resin content, micro-filled resin composites are known to produce the best gloss and surface quality. However, nowadays nano-fill, nano-hybrid and supra-nano-fill resin composites have better surface qualities than micro-filled resin composites [35]. In a study that evaluated the surface quality on universal resin composites which were polished with three different polishing systems which were the same as our polishing systems (Twist Dia, Occlubrush, or Soflex Spiral) found that Occlubrush showed the roughest surface on resin composite and Twist Dia showed the smoothest surface [11]. In another study that tested different polishing systems on the surface roughness of a nanohybrid composite, Twist Dia and Soflex Spiral showed nearly the same surface roughness values, whereas rubber cups showed lower surface roughness values [36].

One of the aesthetic drawbacks of dental resin composites is their extrinsic and/or intrinsic discoloration. Discolored restorations can be fixed by polishing to obtain the original color. The null hypothesis II was accepted. A study in the literature compared the color stability of two universal and one conventional resin composites before and after aging. Coffee and tea were used for staining, and Soflex Spiral wheels were used for polishing. Omnichroma did not give any acceptable result when stained with a green tea solution. In a coffee solution, Optishade did not give acceptable results. The conventional resin composites gave acceptable results in both solutions after aging [37]. In our study, there was no significantly different results between all three polishing systems, except in the group 1 after staining with coffee for 15 days. This may be due to the sample waiting time or the repairment procedure of resin composite samples. In this study, resin composites were immersed in coffee for 15 days. In another study that compared both universal composites, the immersion period was only 24 h. Furthermore, 15 days simulated 1.3 years, whereas 24 h simulated just 1 month [14]. Lastly, since there is a variety of colorants that we might take from foods or drinks, this study may also be supported by other colorant solutions. The last hypothesis, III, was partially accepted. This hypothesis was based on the effect of the repolishing procedure on universal composites after coffee immersion. There were different results between non-polishing and repolishing measurements in all three groups after immersion. According to the polishing system Occlubrush, there was no significant delta value result before and after the polishing procedure. According to the Soflex and Twist polishing systems, there were significant mean values between non-polishing and repolishing procedures in all resin composite groups. This may be caused by the abrasive particles that are included in the polishing system. 

This in vitro study was performed using standard handmade resin composite disc samples that were repaired with resin composites. The study must be also supported with in vivo research. The spectrophotometric measurements must be supported by laboratory measurements also. The bonding system that is used must be supported by other bonding systems. Other universal composite resins used in clinics should also be tested with other colorants. The resin composite base for color measurement was used. It must be supported with black, white and grey bases. 

## 5. Conclusions

Within the limitations of the present study, it was concluded that finishing and polishing systems had significant effects on different universal resin composites, especially after immersion. In general, polishing systems with diamond particles showed significantly better results than polishing systems including aluminum oxide particles or silicon-carbide abrasive particles in removing stains from resin composites stained with coffee. 

Furthermore, universal resin composites showed different potentials for coloring with coffee. Optishade resin composite is useful in clinics according to their group-shade choice. It is easier to use one-shaded resin composite but we found that one-shade resin composite is susceptible to drink colorants. According to the first and third measurement times, Omnichroma was the most stained resin composite. 

The repairment procedure is needed mostly in daily clinic routines. We found that the best way is to repair the resin composites with themselves. However, we found again that when it is necessary to use other resin composites, the second choice must be Optishade. 

## Figures and Tables

**Table 1:** Materials used in study.

Material	Manufacturer	Type	Shade	Abbreviation Used in Tables	Composition
**Omnichroma**	Tokuyama Dental	Universal composite	Single Shade	Omnichroma	UDMA TEGDMA, -Uniform-sized supra-nanospherical filler (260 nm spherical SiO_2_-ZrO_2_) -Composite filler (260 nm spherical SiO_2_-ZrO_2_) -Filler loading 79% by wt. (68% by vol)
**Optishade** **(medium shade)**	Kerr	Universal group-shade composite	Medium	Optishade	BisGMA BisDMA TEGDMA, -Spherical silica and zirconia particles formed from a molecular suspension (effective particle size is 5–400 nm) and 400 nm barium glass particles -Adaptive response technology (ART) with zirconia/silica nanoparticles and rheological modifiers -Filler loading 81% by wt. (64% by vol)
**Clearfil Majesty (Esthetic A2 shade)**	Kuraray	Nano-filled composite	A2	Conventional	Silinated barium glass filler, pre-polymerized organic filler, hydrophobic aromatic dimethacrylate, bis-GMA, di-camphorquinone, other additives
**Twist Dia Spiral Wheels**	Kuraray	Two-step polishing system	-	Twist	Diamond particles
**Sof-Lex Spiral Wheels**	3M ESPE	Two-step polishing system	-	Soflex	Elastomer impregnated with aluminum oxide particles
**Occlubrush**	Kerr	One-step polishing system	-	Occlubrush	Fibers that built-in silicon-carbide abrasive particles
**Palfique Bond**	Tokuyama	Single-component self-etching adhesive	-	Universal bond	Phosphoric acid monomer, bisphenol A di-(2-hydroxy propoxy) dimethacrylate (Bis—GMA), triethyleneglycol dimethacrylate, 2-hydroxyethyl methacrylate (HEMA), camphorquinone, alcohol, and purified water
**Nescafe Gold**	USA	İmmersion material	-	Coffee	Sugar, Instant Coffee, Glucose Syrup, Skim Milk Powder, Coconut Oil, Lactose, Salt, Milk Fat, Flavour, Dipotassium Phosphate, Sodium Polyphosphate, Sodium Citrate

**Table 2:** Distribution of delta values between measurement times (Conventional–Conventional).

Distilled Water	T0–T1	T0–T2	T1–T2
**Ocllubrush**	1.80 ± 0.35 a	2.18 ± 0.35 a	1.69 ± 0.50 a
**Soflex**	1.83 ± 0.63 a	2.83 ± 0.69 a	2.51 ± 0.82 a
**Twist**	2.82 ± 2.01 a	2.40 ± 1.03 a	10.4 ± 1.40 b
**Coffee**	**T0–T1**	**T0–T2**	**T1–T2**
**Occlubrush**	9.82 ± 1.16 a	10.0 ± 1.76 a	5.17 ± 1.27 a
**Soflex**	11.4 ± 2.00 a	5.35 ± 0.88 b	12.0 ± 2.06 b
**Twist**	3.60 ± 1.56 b	1.70 ± 0.55 c	34.7 ± 0.96 c
**Distilled Water–Coffee**	**P^T0–T1^**	**P^T0–T2^**	**P^T1–T2^**
**Occlubrush**	**<0.001**	**<0.001**	**<0.001**
**Soflex**	**<0.001**	**<0.001**	**<0.001**
**Twist**	0.063	0.089	**<0.001**

Mean ± standard deviation values for differences between measurement times of Conventional–Conventional repair. Different lowercase letters indicate the statistically significant differences within the same column (*p* ≤ 0.05).

**Table 3:** Distribution of delta values between measurement times (Conventional–Omnichroma).

Distilled Water	T0–T1	T0–T2	T1–T2
**Occlubrush**	2.70 ± 2.01 a	2.52 ± 0.71 a	5.76 ± 1.04 a
**Soflex**	6.84 ± 10.1 b	3.08 ± 1.19 a	7.85 ± 9.64 a
**Twist**	2.71 ± 1.18 a	2.93 ± 1.34 a	14.5 ± 1.66 b
**Coffee**	**T0–T1**	**T0–T2**	**T1–T2**
**Occlubrush**	15.9 ± 1.98 b	12.1 ± 1.42 b	15.4 ± 2.94 a
**Soflex**	11.2 ± 2.95 a	3.55 ± 1.54 a	12.1 ± 2.88 a
**Twist**	9.17 ± 4.62 a	3.35 ± 2.52 a	31.9 ± 2.63 b
**Distilled Water–Coffee**	**P^T0–T1^**	**P^T0–T1^**	**P^T0–T1^**
**Occlubrush**	**<0.001**	**<0.001**	**<0.001**
**Soflex**	**<0.001**	**<0.001**	**<0.001**
**Twist**	0.063	0.063	0.063

Mean ± standard deviation values for differences between measurement times of Conventional–Omnichroma repair. Different lowercase letters indicate statistically significant differences within the same column (*p* ≤ 0.05).

**Table 4:** Distribution of delta values between measurement times (Conventional–Optishade).

Distilled Water	T0–T1	T0–T2	T1–T2
**Occlubrush**	2.01 ± 1.10 a	1.70 ± 0.47 a	2.71 ± 2.74 a
**Soflex**	3.33 ± 0.48 b	4.17 ± 2.64 b	1.86 ± 0.94 a
**Twist**	1.23 ± 0.34 a	2.08 ± 1.17 a	11.0 ± 1.06 b
**Coffee**	**T0–T1**	**T0–T2**	**T1–T2**
**Occlubrush**	13.3 ± 2.83 b	10.9 ± 1.49 b	11.1 ± 2.85 a
**Soflex**	9.71 ± 2.44 a	2.95 ± 1.10 a	8.91 ± 2.07 a
**Twist**	5.24 ± 2.61 a	1.38 ± 0.36 a	34.7 ± 1.42 b
**Distilled Water-Coffee**	**P^T0–T1^**	**P^T0–T2^**	**P^T1–T2^**
**Occlubrush**	**<0.001**	**<0.001**	**<0.001**
**Soflex**	**<0.001**	0.280	**<0.001**
**Twist**	**<0.001**	0.315	**<0.001**

Mean ± standard deviation values for differences between measurement times of Conventional–Optishade repair. Different lowercase letters indicate statistically significant differences within the same column (*p* ≤ 0.05).

**Table 5:** Analysis of delta values between Polishing Types (Conventional–Conventional).

Distilled Water	Occlubrush–Soflex	Soflex–Twist	Occlubrush–Twist
**T0**	1.75 ± 0.45 a	2.03 ± 0.40 a	1.10 ± 0.53 b
**T1**	1.31 ± 0.59 a	2.94 ± 1.49 a	2.50 ± 1.73 a
**T2**	2.12 ± 0.79 b	1.61 ± 1.10 b	2.57 ± 1.17 a
**Coffee**	**Occlubrush–Soflex**	**Soflex–Twist**	**Occlubrush–Twist**
**T0**	1.25 ± 0.28 b	1.52 ± 0.47 b	0.92 ± 0.42 b
**T1**	3.87 ± 2.34 a	9.70 ± 2.31 a	7.49 ± 1.78 a
**T2**	5.29 ± 1.94 a	5.55 ± 1.27 a	10.0 ± 1.66 a
**Disttilled Water-Coffee**	**p ^Occlubrus–Soflex^**	**p ^Soflex–Twist^**	**p ^Occlubrush–Twist^**
**T0**	**0.011**	0.075	0.436
**T1**	**0.002**	**<0.001**	**<0.001**
**T2**	**<0.001**	**<0.001**	**<0.001**

Mean ± standard deviation values for different values between polishing types for Conventional–Conventional repair. Different lowercase letters indicate statistically significant differences within the same column (*p* ≤ 0.05).

**Table 6:** Analysis of delta values between Polishing Types (Conventional–Omnichroma).

Distilled Water	Occlubrush–Soflex	Soflex–Twist	Occlubrush–Twist
**T0**	3.58 ± 1.05 a	2.43 ± 1.42 a	4.91 ± 0.94 a
**T1**	7.63 ± 11.0 a	7.51 ± 9.79 a	5.24 ± 0.56 a
**T2**	4.02 ± 0.96 a	1.83 ± 1.21 b	4.44 ± 0.85 a
**Coffee**	**Occlubrush–Soflex**	**Soflex–Twist**	**Occlubrush–Twist**
**T0**	2.59 ± 1.17 b	3.69 ± 1.69 a	4.78 ± 0.73 b
**T1**	6.96 ± 3.30 a	6.38 ± 4.03 a	10.9 ± 5.95 a
**T2**	10.9 ± 1.93 a	4.01 ± 2.07 a	13.5 ± 1.59 a
**Distilled Water-Coffee**	**p ^Occlubrush–Soflex^**	**p ^Soflex–Twist^**	**p ^Occlubrush–Twist^**
**T0**	0.075	0.075	0.912
**T1**	0.165	0.529	**0.023**
**T2**	**<0.001**	**0.003**	**<0.001**

Mean ± standard deviation values for differences between polishing types for Conventional–Omnichroma repair. Different lowercase letters indicate statistically significant differences within the same column (*p* ≤ 0.05).

**Table 7:** Analysis of delta values between Polishing Types (Conventional–Optishade).

Distilled Water	Occlubrush–Soflex	Soflex–Twist	Occlubrush–Twist
**T0**	2.70 ± 0.55 b	2.34 ± 0.52 a	1.23 ± 0.74 a
**T1**	1.67 ± 1.07 a	0.87 ± 0.54 b	1.84 ± 0.87 a
**T2**	1.80 ± 3.00 a	2.13 ± 2.89 a	1.67 ± 0.92 a
**Coffee**	**Occlubrush–Soflex**	**Soflex–Twist**	**Occlubrush–Twist**
**T0**	1.09 ± 0.70 b	2.02 ± 1.24 a	1.94 ± 1.11 b
**T1**	4.52 ± 3.02 a	5.84 ± 1.97 a	8.53 ± 3.69 a
**T2**	8.73 ± 1.71 a	1.53 ± 1.17 b	9.96 ± 1.13 a
**Distilled Water–Coffee**	**p ^Occlubrush–Soflex^**	**p ^Soflex–Twist^**	**p ^Occlubrush–Twist^**
**T0**	**<0.001**	0.739	0.165
**T1**	**0.003**	**<0.001**	**<0.001**
**T2**	**<0.001**	1.00	**<0.001**

Mean ± standard deviation values for differences between polishing types for Conventional–Optishade repair. Different lowercase letters indicate statistically significant differences within the same column (*p* ≤ 0.05).

**Table 8:** Analysis of delta values between Resin Composite Measurements.

Conventional–Conventional	Occlubrush	Soflex	Twist
**T0**	0.89 ± 0.52 bX	1.08 ± 0.35 aX	0.79 ± 0.38 aX
**T1**	9.57 ± 1.08 aX	11.4 ± 2.08 bX	4.08 ± 1.73 bY
**T2**	9.62 ± 2.30 aX	6.36 ± 0.72 cX	1.73 ± 1.13 aY
**Conventional–OMNI**	**Occlubrush**	**Soflex**	**Twist**
**T0**	1.27 ± 0.35 bY	2.90 ± 0.96 aX	2.03 ± 1.70 aX
**T1**	15.5 ± 1.76 aX	14.2 ± 8.80 bX	9.00 ± 4.45 bY
**T2**	13.2 ± 1.76 aY	4.57 ± 1.68 aX	3.00 ± 1.23 aX
**Conventional–OPTI**	**Occlubrush**	**Soflex**	**Twist**
**T0**	1.97 ± 1.05 bY	1.75 ± 0.72 aX	0.72 ± 0.25 aX
**T1**	13.3 ± 2.48 aY	9.35 ± 2.33 bX	5.53 ± 2.61 bX
**T2**	10.6 ± 0.96 aY	3.69 ± 2.39 cX	2.26 ± 1.01 cX

Mean ± standard deviation values for differences between polishing types for different resin composite repairs. Different lowercase letters indicate statistically significant differences within the same column (*p* ≤ 0.05) (between measurement times). Different capital letters indicate statistically significant difference in the same row (between polishes) (*p* ≤ 0.05).

**Table 9:** ΔL, ΔC, ΔH values of samples.

COMPOSITE REPAIRMENT MATERIAL	POLISHING TYPE	MEASUREMENT TIME	ΔL	ΔC	ΔH
**CLEARFIL MAJESTY ESTHETIC (A2** **SHADE)** **(GROUP 1)**	Occlubrush	T0	0.09	0.15	−0.04
		T1	8.89	−18.39	7.68
		T2	10.76	−13.98	9.94
	Soflex Spiral Wheels	T0	−1.37	0.36	−0.29
		T1	7.64	−19.66	10.2
		T2	6.96	−9.27	9.54
	Twist Dia Wheels	T0	0	−0.42	0.32
		T1	4.66	−5.39	−0.34
		T2	2.83	−5.21	−2.69
**OMNICHROMA** **(ONE-SHADE)** **(GROUP 2)**	Occlubrush	T0	−0.65	−0.07	−0.98
		T1	15.18	−22.79	16.59
		T2	10.49	−14.83	27.84
	Soflex Spiral Wheels	T0	3.34	−0.98	3.33
		T1	23.93	−15.3	18.51
		T2	4.68	−5.19	11.61
	Twist Dia Wheels	T0	−0.27	1.08	−5.61
		T1	10.16	−10.07	17.45
		T2	4.5	−1.24	13.45
**OPTISHADE** **(MEDIUM SHADE)** **(GROUP 3)**	Occlubrush	T0	−1.04	0.18	−3.54
		T1	18.5	−22.99	12.87
		T2	14.39	−16.89	7.79
	Soflex Spiral Wheels	T0	0.8	−0.45	−0.95
		T1	5.22	−16.91	6.64
		T2	1.34	−5.19	0.98
	Twist Dia Wheels	T0	0.28	−0.08	0.69
		T1	7.89	−10.07	6.26
		T2	2.65	−3.32	0.06

## Data Availability

The data presented in this study are available on request from the corresponding author. The data are not publicly available due to security.
